# Characterization of the differentially methylated region of the *Impact *gene that exhibits Glires-specific imprinting

**DOI:** 10.1186/gb-2008-9-11-r160

**Published:** 2008-11-13

**Authors:** Kohji Okamura, Richard F Wintle, Stephen W Scherer

**Affiliations:** 1 The Centre for Applied Genomics, Program in Genetics and Genome Biology, The Hospital for Sick Children, MaRS Centre TMDT, 101 College Street, Toronto, Ontario M5G 1L7, Canada; 2 Department of Molecular and Medical Genetics, University of Toronto, Toronto, Ontario M5S 1A8, Canada; 3 Current address: Human Genome Centre, Institute of Medical Science, University of Tokyo, 4-6-1 Shirokanedai, Minato Ward, Tokyo 108-8639, Japan

## Abstract

**Background:**

Imprinted genes are exclusively expressed from one of the two parental alleles in a parent-of-origin-specific manner. In mammals, nearly 100 genes are documented to be imprinted. To understand the mechanism behind this gene regulation and to identify novel imprinted genes, common features of DNA sequences have been analyzed; however, the general features required for genomic imprinting have not yet been identified, possibly due to variability in underlying molecular mechanisms from locus to locus.

**Results:**

We performed a thorough comparative genomic analysis of a single locus, *Impact*, which is imprinted only in Glires (rodents and lagomorphs). The fact that Glires and primates diverged from each other as recent as 70 million years ago makes comparisons between imprinted and non-imprinted orthologues relatively reliable. In species from the Glires clade, *Impact *bears a differentially methylated region, whereby the maternal allele is hypermethylated. Analysis of this region demonstrated that imprinting was not associated with the presence of direct tandem repeats nor with CpG dinucleotide density. In contrast, a CpG periodicity of 8 bp was observed in this region in species of the Glires clade compared to those of carnivores, artiodactyls, and primates.

**Conclusions:**

We show that tandem repeats are dispensable, establishment of the differentially methylated region does not rely on G+C content and CpG density, and the CpG periodicity of 8 bp is meaningful to the imprinting. This interval has recently been reported to be optimal for *de novo *methylation by the Dnmt3a-Dnmt3L complex, suggesting its importance in the establishment of imprinting in *Impact *and other genes.

## Abstract 2

Comparative genomic analysis of the *Impact* locus, which is imprinted in Glires but not in other mammals, reveals features required for genomic imprinting.

## Background

Genomic imprinting is an epigenetic modification that leads to monoallelic gene expression in a parent-of-origin-specific manner. In mammals, approximately 100 'imprinted' genes are subject to this regulation [1]. Identification of a specific sequence that is recognized as the target for epigenetic marking is the foremost problem in this field. Researchers have compared genomic sequences of human and mouse imprinted and non-imprinted genes in order to identify motifs that are characteristic of, or responsible for, genomic imprinting [2-5]. Especially, finding target sequences for *de novo *DNA methylation during gametogenesis would further our understanding of the molecular mechanisms of imprinting, as well as development, tissue-specific gene regulation, and the etiology of various cancers. However, genomic features unique to imprinted genes, which could lead to their discovery, have not been described, with one exception [6]. It has been suggested that the absence of such features is due to variability in the molecular mechanisms of imprinting [7,8].

Therefore, instead of identifying common features, we limited our study to one imprinted gene, *Impact*, but performed comparative genomics among thirty eutherian species. The *Impact *gene was first identified in mouse as a novel imprinted gene by a systematic screening method using mRNA display PCR [9]. Its protein product is suggested to have a role in response to amino acid starvation [10,11]. This gene exhibits species-specific imprinting; it is imprinted in species of the Glires clade (rodents and lagomorphs), but not in other mammals such as primates and artiodactyls (even-toed ungulates) [12]. Since the Glires clade diverged from primates approximately 70 million years ago [13], the acquisition of the imprinting in these species is quite recent compared to other imprinted genes, most of which are imprinted in both mouse and human. This makes the comparative analysis between imprinted and non-imprinted orthologues more straightforward. By contrast, if we studied, for example, the *Igf2 *gene by the same strategy, we would have to compare two clades, for example, eutherians and monotremes, which diverged about 200 million years ago [14]. Generally, such sequences are too divergent to allow DNA motifs to be found by sequence alignment. The recent evolution of *Impact *as an imprinted gene provides a unique opportunity to perform this kind of comparative genomics.

In species of the Glires clade, *Impact *bears a differentially methylated region (DMR) in its first intron that is *de novo *methylated during oogenesis, but not in spermatogenesis, and maintained in all types of somatic cells to adulthood [15]. Hence, this region is a so-called primary DMR, which is the key *cis*-regulatory element directing the correct establishment and maintenance of genomic imprinting. In our previous analysis of the *Impact *DMR in species of the Glires clade, the sequences of mouse, rat, and rabbit were determined. The DMR in these species is characterized by a CpG island, and the DMR in rodents contains characteristic tandem repeats in the CpG island [12]. Because the mechanism by which the *de novo *DNA methylation machinery recognizes the DMRs is not yet known, we have tried in the present study to search for the target sequences of the allele-specific methylation by sequencing the genomic region of various Glires animals, including beaver, porcupine, chipmunk, and prairie dog. Fortunately, the first intron could readily be amplified by PCR using primers located in the first and second exons. Including data from our previous study [12], 27 out of 30 eutherian species were successfully sequenced.

More than a decade ago, direct tandem repeats were suggested to be related to genomic imprinting [16]; however, the numbers of identified imprinted genes and available mouse and human genomic sequences were considerably limited at that time. Later, *Impact *was identified, and it was reported that imprinted mouse *Impact *bears these characteristic repeats whereas the non-imprinted human orthologue lacks any apparent repeats [17]. It was subsequently reported that the repeat is absent in the imprinted rabbit *Impact *gene [12]. Since tandem repeats are abundant and widespread throughout mammalian genomes [18], it is therefore difficult to associate these with the imprinting status of specific genes. One strategy to address this is to increase the number of species studied at a given locus. A recent study determining the extent and boundaries of all known primary DMRs enabled the analysis of their specific nucleotide sequences and content [19]. Some characteristic features were described; however, the number of primary DMRs in mouse is limited to only 15 to date. Our study provides additional data that are needed to characterize such intriguing regions.

In support of the fast molecular clock of rodent genomes [20], we observe that the determined genomic sequences are considerably diverged only among rodents, but not in lagomorphs. While the data challenge the proposed role of tandem repeats and CpG content in genomic imprinting, they suggest the importance of latent CpG dinucleotide periodicity in the establishment of the *Impact *DMR.

## Results

We previously developed a simple PCR-based strategy to determine the nucleic acid sequence of the first intron of *Impact *and reported the sequences of 14 eutherian species [12]. In this method, primers were designed for highly conserved regions in exons 1 and 2 for forward and reverse primers, respectively. Two forward and two reverse degenerate primers were prepared to perform nested PCR for the divergent sequences. In the present study, we used the same method to determine the corresponding sequences in two lagomorphs and 17 rodents (Table 1). All but three were successfully amplified. For these species (field mouse, agouti, and paca), specific PCR products could not be obtained even after nested PCR. This is probably due to unexpectedly divergent sequences at the exonic priming sites or excessive elongation of the intron in these animals (see Discussion).

**Table 1:** Lagomorphs and rodents used in this study

Species	Taxonomy ID*	Common name^†^	Accession number
*Oryctolagus cuniculus*	9986	Rabbit	EF470590
*Sylvilagus floridanus*	9988	Cottontail	EF470591
*Peromyscus maniculatus*	10042	Deer mouse	EF470592
*Mus musculus*	10090	Mouse	EF470593
*Rattus norvegicus*	10116	Rat	EF470594
*Erethizon dorsatum*	34844	Porcupine	EF470595
*Hylomyscus alleni*	34858	Wood mouse	EF470596
*Apodemus agrarius*	39030	Field mouse	-
*Dasyprocta leporina*	42152	Agouti	-
*Cynomys ludovicianus*	45480	Prairie dog	EF470597
*Castor canadensis*	51338	Beaver	EF470598
*Rhizomys pruinosus*	53275	Bamboo rat	EF470599
*Tamias sibiricus*	64680	Chipmunk	EF470600
*Eothenomys melanogaster*	82468	Vole	EF470601
*Dicrostonyx groenlandicus*	85953	Lemming	EF470602
*Agouti paca*	108852	Paca	-
*Reithrodontomys gracilis*	243215	Harvest mouse	EF470603
*Tscherskia triton*	329627	Hamster	EF470604
*Rheomys thomasi*	451894	Water mouse	EF470605

*Assigned by NCBI Taxonomy. ^†^Common names used in this paper. Some of these are short forms of GenBank common names, such as American beaver. See NCBI Taxonomy.

Following treatment with exonuclease I and shrimp alkaline phosphatase, nested PCR products were directly sequenced by the primer-walking method. The identities of these amplicons as the *Impact *gene were confirmed by the 30-nucleotide sequences at the beginning of exon 2. This short region was also amplified along with the first intron for this purpose. Almost all encode an amino acid sequence identical to NEEIEAMAAI seen in human IMPACT. Exceptions were mouse, wood mouse, bamboo rat, and porcupine, which code for SEEIEAMAAI, SEEIEAMAAI, NEEIEAMASI, and NEEIEALSAI, respectively. It has been surmised that *Impact *does not have paralogues in any vertebrate genome due to dosage sensitivity [21]. Accordingly, a PCR product amplified from a single locus was obtained in each species. We also confirmed that all of the intronic sequences meet the GT-AG rule, also known as Chambon's rule, and that they have a branch site proximal to the splice acceptor (not shown).

**Figure 1:**
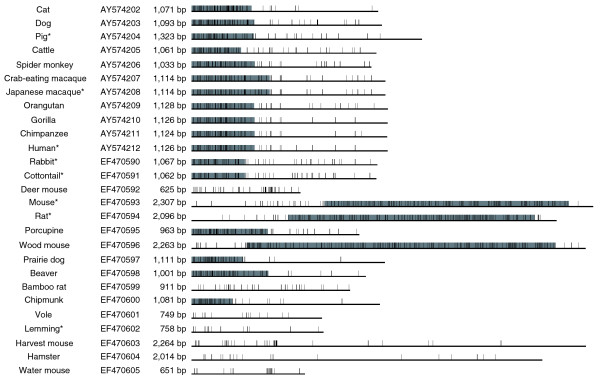
Schematic representation of the first intron of eutherian *Impact*. The GenBank accession number and length are listed to the right of the common names. Horizontal lines show the relative lengths of the first intron. All sequences begin with GT and end with AG. Short vertical lines and gray boxes represent single CpG sites and CpG islands, respectively, which were detected by GrailEXP 3.31. Characteristic tandem repeats are exclusively found in the CpG islands of murids (mouse, rat, and wood mouse). Glires species are sorted by NCBI Taxonomy ID. The *Impact *gene is assumed to be imprinted in Glires species [GenBank:EF470590-EF470605] but not in other species [GenBank:AY574202-AY574212]. Asterisks indicate species whose monoallelic expression or methylation of the *Impact *gene have been experimentally confirmed [12,17].

In the previous study using rodents, lagomorphs, artiodactyls, carnivores, and primates, the sequences were readily classified into two groups (Figure 1). The first group has a longer intron (approximately 2 kb), the 3' portion of which constitutes a CpG island with a characteristic tandem reiterated structure [17]. The second group has a shorter intron (approximately 1 kb), the 5' portion of which constitutes a short CpG island without any apparent repeats. Regardless of the imprinting status of the *Impact *gene, only mouse and rat sequences fall in the former group. Despite the fact that rabbit *Impact *is imprinted, it was unexpectedly categorized in the latter group. Additionally, a sequence derived from the whole genome shotgun sequencing of the rabbit was obtained [GenBank:AAGW01108706], which covers this region and confirms the absence of tandem repeats, even in the expanded flanking regions included in this sequence. In mouse, the two genes flanking *Impact *are not imprinted and no additional imprinted genes have been found on chromosome 18 where it is mapped [22]. Unlike typical imprinted genes, *Impact *appears to be solitary; it is likely that the regulatory elements are confined to this locus. Hence, at least for this imprinted locus, the result clearly negates a hypothesis that tandem repeats play an important role in genomic imprinting [16]. To pursue other structural features of imprinted *Impact*, elucidating the genomic sequences of many other rodent and lagomorph species was of interest.

**Figure 2:**
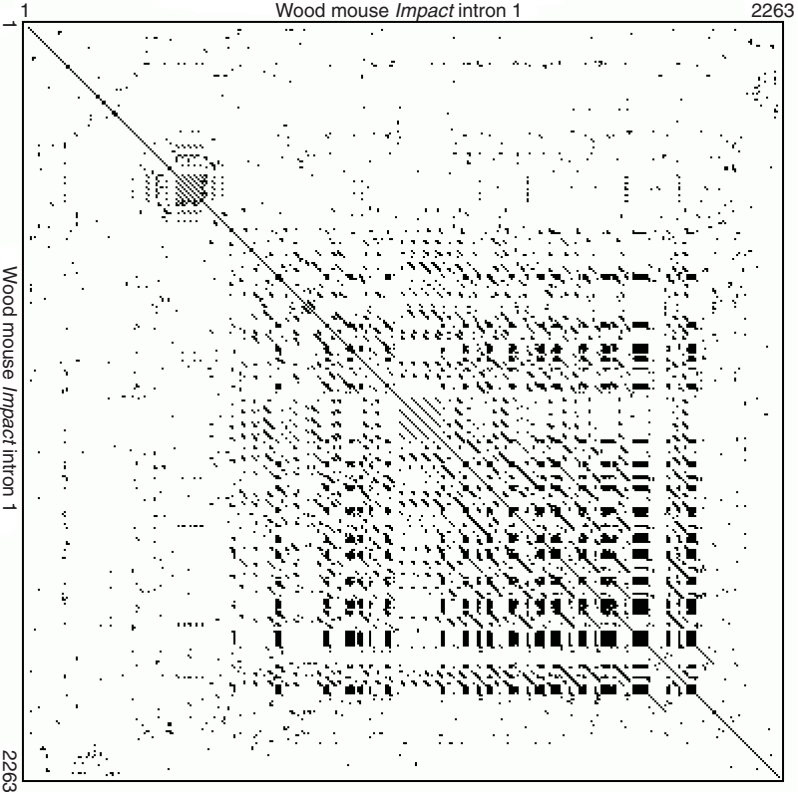
Direct tandem repeat of wood mouse *Impact*. Self-Harr plot of the first intron of wood mouse *Impact *shows nested structure of direct tandem repeats around the CpG island. A dot was plotted when it satisfied the condition that there were more than 8 bases matching in a 10-bp window. While mouse and rat *Impact *also show quite similar plots, other eutherians apparently do not have this tandem repeat.

The genomic sequences determined in the current study are shown along with previous results (Figure 1). While lagomorphs (rabbit and cottontail) have similar intronic sequences to those of primates, artiodactyls, and carnivores, rodents have diversified structures. Although the porcupine, beaver, and sciurids (prairie dog and chipmunk) bear a CpG island at the 5' end like lagomorphs, murids (mouse, rat, and wood mouse) bear a longer one at the 3' side. Others unexpectedly bear no CpG islands. The lengths of these introns vary from 625 bp to more than 2 kb. The characteristic tandem repeat was found exclusively in murids (Figure 2). A homology search using the repetitive regions as queries did not hit any other sequences but themselves, suggesting that these sequences are unique to this locus in murids.

**Figure 3:**
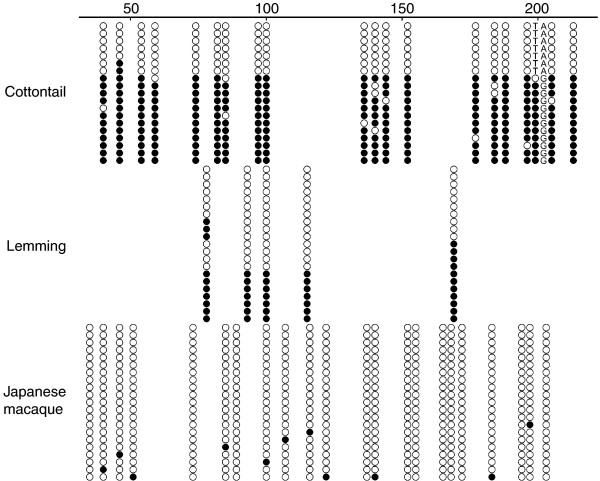
DNA methylation analysis by bisulfite cloning and sequencing. The analysis was carried out for three species. Cottontail is one of the lagomorphs. Lemming (a rodent) contains fewer CpG sites in the first intron of *Impact*. Japanese macaque is a primate in which the *IMPACT *gene is not imprinted. Numbers indicate distances in base-pairs from the 3' end of the first exon. Each row represents an individual cloned allele. Circles represent CpG sites and their spacing reflects the CpG density of the region. Filled and open circles represent methylated and unmethylated sites, respectively. The single nucleotide polymorphism in cottontail fortunately provided allele-specific methylation data, although the parental origin is unknown. Note that this single nucleotide substitution has caused a coexistence of TpG and CpG; only the latter is subject to deamination.

The scarcity of CpG dinucleotides in several rodents made us wonder whether they bear the DMR in this region and whether they are imprinted or not. We therefore chose lemming as one of those species, cottontail from lagomorphs, and Japanese macaque from the non-imprinted group for DNA methylation analysis by bisulfite cloning and sequencing [23]. For both mouse and rabbit *Impact*, the 5' portion of the first intron was shown to be subject to allele-specific methylation; the maternal and paternal alleles are hyper- and hypomethylated, respectively [12,17,19]. We decided to analyze the equivalent region for these three species (Figure 3).

We used one individual from each species. Fortunately, the cottontail has one A/G heterozygous site (position 201 of the sequence deposited under [GenBank:EF470591]) in this region, which allowed us to distinguish the two alleles. Although the parental origin could not be ascertained, one of the parental alleles is unmethylated and the other is heavily methylated. Possibly, the paternal allele of cottontail *Impact *may be exclusively expressed like rabbit *Impact *[12]. Unlike cottontail *Impact*, the lemming gene has only five CpG sites with no heterozygous sites in this region. However, the result suggests that the region is a DMR because there were unmethylated clones and fully methylated clones. It is likely that lemming *Impact *is also imprinted like other rodent orthologues despite the scarceness of CpG dinucleotides in the corresponding region. Macaque *IMPACT *has a CpG island in this region like the cottontail gene. In support of the fact that primate *Impact *exhibits biallelic expression [12], the 5' portion of the intron escapes DNA methylation in both alleles in Japanese macaque. Establishment of the DMR seems to be independent, not only of tandem repeats, but also of local CpG density. This raises another question: what then causes the difference in DNA methylation status between Glires and other mammals?

**Figure 4:**
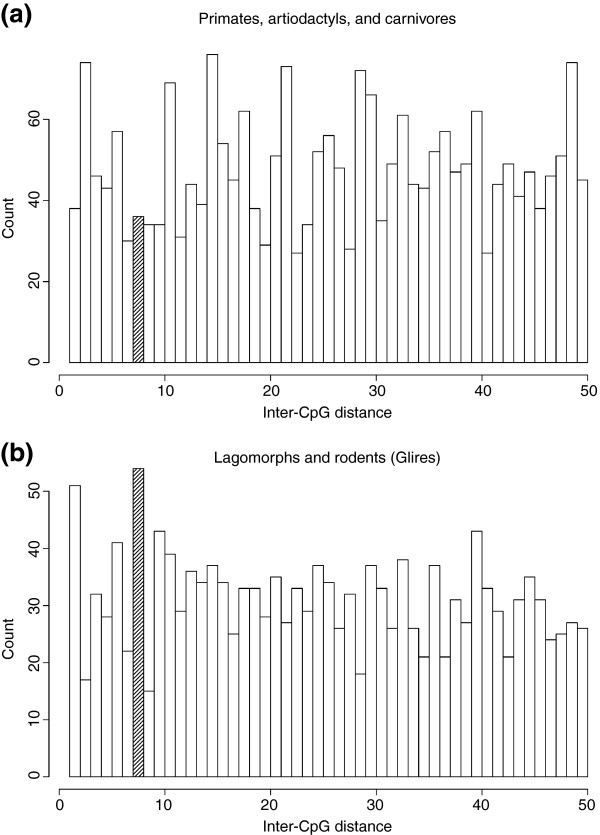
Periodicity of CpG sites in the 500-bp region at the 5' portion of the intron. Counts of each distance from 2-50 bp are shown for **(a) **non-Glires eutherians and **(b) **Glires species. The 8-bp periodicity is evident only in Glires (*p *= 2.46 × 10^-3^; see Materials and methods), which bears the DMR in this region.

Recently, crystallography of a complex consisting of Dnmt3a and Dnmt3L revealed a correlation between its enzymatic activity and methylated CpG sites at distances of eight to ten base pairs [24]. Dnmt3a is a DNA methyltransferase and Dnmt3L is its regulatory factor; both of these proteins are needed for the *de novo *DNA methylation of imprinted genes [25-27]. Accordingly, periodicity of CpG dinucleotide locations is found in the DMRs of 12 imprinted genes that are subject to maternal methylation. Mouse *Impact *is one of these genes, bearing a large number of CpG dinucleotides spaced with 10-bp periodicity [24]. However, this periodicity originates in the direct repeats found only in murids. In order to search for other CpG periodicity that may be related to the *de novo *DNA methylation of the *Impact *DMR, we examined only the 500-bp region at the 5' end of the intron in the eutherians. Frequencies of CpG pairs at a given distance with respect to all pairs are separately shown for Glires species (putative imprinted group) and other eutherians in Figure 4. While a conspicuous 8-bp CpG interval, but neither a 7- nor 9-bp interval, is observed in species of the Glires clade, the frequency of 8-10-bp intervals in other eutherian species is low (*p *= 2.46 × 10^-3^; see Materials and methods). Additionally, the periodic occurrence of CpG sites 9.5 bp apart on average was not observed in this region [24] (Additional data file 1). These results suggest that the CpG periodicity of 8 bp plays an important role in imprinting and that the accumulation of this periodicity might relate to acquisition of imprinting in the common ancestor of extant Glires species.

## Discussion

Whereas the possible importance of tandem repeats in genomic imprinting is still disputed [28-30], several lines of evidence negate the hypothesis [31-34]. The present study also argues against the proposed role of repetitive elements in the imprinting of *Impact*. Since it is suggested that imprinting has evolved randomly at various times in different lineages [7], molecular mechanisms that achieve monoallelic gene expression may vary from locus to locus. Tandem repeats can be observed almost everywhere in mammalian genomes [18]. Hence, it seems unreasonable to assume that tandem repeats *per se *have a role in genomic imprinting in general. What we should address is a specific role of each tandem repeat, such as offering a high concentration of insulator binding sites [35], rather than presence or absence of any repeats.

The tandem repeat in murid *Impact *has a complex structure with nested repetitive elements, but the shortest sequence element is 5'-TCGGC-3'. This 5-bp directed element is concatenated to constitute the long stretch in mouse, rat, and wood mouse genomes. It is possible that 10-bp periodicity, which is caused by juxtaposition of the element, is so stable for nucleosome positioning that it allows the region to expand the repeat. It is reported that 10-bp periodic GpC, which corresponds to one DNA helical repeat, is often found in regions that form nucleosome structure well [36]. The shortest element definitely contains GpC dinucleotide (note that this is not CpG dinucleotide). It is also likely that tandem repeats near imprinted genes are just a consequence, rather than a cause, of the epigenetic regulation [37]. The 3' portion of the CpG island appears to be just such a product of expansion of an element containing a single CpG, resulting in high frequency of CpG. This region in the field mouse, another murid, failed to amplify by PCR. Possibly, a large repeat expansion in the intron impedes the PCR amplification of the field mouse genome; however, we have not tested this. Similarly, neither agouti nor paca, closely related caviomorph rodents, could be amplified by PCR at this locus. Possibly, they have a unique shared substitution or insertion that prevented amplification. The chicken intron also could not be amplified by this method.

It was suggested that CpG content *per se *could be recognized by methylation machinery to give rise to primary DMRs [19]. Contrary to this hypothesis, the CpG content in the *Impact *DMR turned out to vary considerably among species of the Glires clade, also suggesting necessity to search for DMRs other than CpG islands. Rather than discern the CpG dinucleotide density, the *de novo *methylation complex seemingly prefers to interact with CpG sites arranged at an interval of 8 bp. The 8-bp CpG periodicity was preferentially observed in Glires, in which the *Impact *gene is imprinted (Figure 4). In a broad sense, the periodicity 5'-CGNNNNNNCG-3' can be considered as a DNA motif or protein-biding site that is targeted by the Dnmt3a-Dnmt3L complex. It is possible that accumulation of the motif in the common ancestor of Glires was related to the acquisition of the *Impact *imprinting. In fact, the short genomic sequence of lemming shown here does not contain the 8-bp periodicity. We do not insist that the periodicity is the necessary and sufficient factor for the genomic imprinting; however, it seems to have a role (Figure 4 and Additional data file 1).

One possible hypothesis is that, in the common ancestor, tandem duplication of a short fragment containing 8-bp CpG periodicity occurred repeatedly, resulting in recruitment of methylation machinery during oogenesis. In this model, critical sites for the interaction with the enzymatic complex are CpG dinucleotides at an interval of 8 bp. The other nucleotides could have been neutrally mutated or diverged because the change does not affect the DNA-protein interaction. In any case, the present study also suggests a limit to the usefulness of conventional homology search algorithms for detecting imprinted genes. It may be important to investigate unexplored features of genomic sequences like the latent periodicity suggested by our studies. Each *de novo *DNA methyltransferase seems to have a specific genomic context associated with methylation, although functional redundancy is also observed [38]. In our additional analysis of the mouse genome, obvious, moderate, and much lower 8-bp periodicities were observed in SineB1, IAP, and Line1 repeats, respectively (data not shown; see Materials and methods). These results seem consistent with the experiment using *Dnmt3*-mutant mice [38]. The most parsimonious explanation is that the 12 maternally methylated DMRs are methylated by the same protein complex. By this expanded comparative analysis, we could successfully exclude the potential role of the 10-bp periodicity in the *Impact *imprinting described above [24]. For the other 11 DMRs, further analysis of the kind presented here may facilitate the understanding of genomic imprinting. Considering the molecular mechanisms that are needed, characteristic features of genomic sequences in imprinted genes should be identified in order to elucidate the true nature of genomic imprinting.

## Conclusions

As a step towards a better understanding of the establishment of DMRs, we took the unique approach of using comparative genomics. Only one species-specific imprinted gene was chosen, but various mammalian genomic DNAs were collected. The results are summarized by the following three points. First, direct tandem repeats, which are found only in murids, are dispensable for the imprinting. Second, establishment of the DMRs does not rely on of G+C content and CpG density. Finally, a CpG periodicity of 8 bp, but neither 9 nor 10 bp, may play an important role in the establishment of this imprinting. Serial duplication of this region could have resulted in the accumulation of this periodicity, which might be related to establishment of imprinting at this locus in the common ancestor of rodents and lagomorphs. These three are apparently true at least for the *Impact *gene. Nevertheless, the method and implication documented in the present study should be applied to many other loci in order to help understand the general molecular mechanisms of genomic imprinting.

## Materials and methods

### Animal resources

Rodent and lagomorph tissues (livers or spleens) were generous gifts from the Royal Ontario Museum (ROM) in Toronto, Ontario, Canada. Rabbits and rats were derived from closed colonies maintained by Kitayama Labes (Ina, Nagano, Japan) and Clea Japan (Tokyo, Japan), respectively. The Japanese macaque (*Macaca fuscata*) brain was a gift from Dr Hiroyuki Okuno at University of Tokyo.

### Sequencing the first intron of *Impact*

Genomic DNA was extracted from livers or spleens of rodents and cottontail, and from brains of a rabbit, rat, and macaque. The first round of PCR was performed using primers 5'- ATG GCT GAR GDG GAM KYA GGG A -3' (forward) and 5'- CAA AGT GTC CAT TTG GGG TCA TC -3' (reverse). The second round of PCR was performed using a pair of nested primers: 5'- AGG GAR CRR CCA GAG GCA G -3' (forward) and 5'- ACA CAC CAC TCC TCG CCA TA -3' (reverse). Both PCR reactions were performed in the presence of 3.5% dimethyl sulfoxide (DMSO). PCR products were treated with exonuclease I and shrimp alkaline phosphatase (Amersham, London, UK) for subsequent direct sequencing. Sequence data from this article has been deposited as [GenBank:EF470590-EF470605].

### DNA methylation analysis

We used the EpiTect Bisulfite Kit (Qiagen, Germantown, MD, USA) for the bisulfite treatment of genomic DNA. Primers used for lemming, cottontail, and macaque were 5'- GTG AGG TTT TTY GGG TAG GGA AYG G -3' (forward), 5'- CAA TAA ACT CCA AAC CAA CCA CAA C TAT AC -3' (reverse), 5'- GTG AGG TTT YGG YGG GGY GTT GTT -3' (forward), 5'- CTA CCT ACA ACC CAC TAC TAC TCA ATC -3' (reverse), 5'- GTG AGG TTT YGG YGG GGT GTT GAT -3' (forward), and 5'- CAC CRT CCR AAA CAA ACC CAA CCC -3' (reverse), respectively. For each species, the amplified positions are 1-224, 1-240, and 1-227, respectively. Position 1 corresponds to the first nucleotide (the 5' end) of the intron and also to the position in the GenBank data.

### Computational analysis of DNA sequences

CpG islands were detected with GrailEXP 3.31 [39]. Mouse repetitive elements, that is, SineB1, IAP, and Line1, were identified by RepeatMasker Open-3.1.9 using a modified library [40]. Other analyses, such as showing each CpG site and determining the frequencies of intervals between two CpG dinucleotide sites, were performed using Perl scripts, which are available upon request from KO.

### Statistical tests for CpG periodicity

We evaluated the statistical significance of the periodicities between imprinted and nonimprinted groups at distances from 2-50 bp using the one-tailed Fisher's exact test. We also employed the Bonferroni method for multiple testing correction of the *p*-values estimated from the tests [41]. Among distances from 2-50 bp, 8 bp is the only periodicity that has a significantly higher count in the imprinted group than in the nonimprinted group at a significance level of 0.01 (Additional data file 1).

## Abbreviations

DMR, differentially methylated region.

## Authors' contributions

KO conceived of the study, performed experiments, analyzed data, and drafted the manuscript. RFW and SWS participated in the coordination of the study, interpretation of data, and helped draft the manuscript. All authors had the opportunity to discuss the results and comment on the final manuscript.

## Additional data files

The following additional data are available. Additional data file 1 is a table showing the numeric data used to draw Figure 4 and the *p*-values of Fisher's exact test and Bonferroni correction for the periodicity of CpG sites.

## Supplementary Material

Additional data file 1 Numeric data used to draw Figure 4 and the *p*-values of Fisher's exact test and Bonferroni correction for the periodicity of CpG sites. Click here for file
