# Supplementary material for: Characterization of the differentially methylated region of the Impact gene that exhibits Glires-specific imprinting
Source: Genome Biol. 2008 Nov 13;9(11):R160. doi: 10.1186/gb-2008-9-11-r160 (PMC2614492; doi:10.1186/gb-2008-9-11-r160)
Supplement: Additional data file 1 — Numeric data used to draw Figure 4 and the p-values of Fisher's exact test and Bonferroni correction for the periodicity of CpG sites. [file gb-2008-9-11-r160-S1.pdf]

Additional data file 1. One-tailed Fisher's exact test and Bonferroni correction for the periodicity of CpG sites

Distance	Count at a given distance in the imprinted group	Count at a given distance in the nonimprinted group	Count at other distances in the imprinted group	Count at other distances in the nonimprinted group	Fisher's exact test *p*-value	Bonferroni correction *p*-value
2	51	38	1473	2309	4.0561E-04	0.0199
3	17	74	1507	2273	1.0000	1.0000
4	32	46	1492	2301	0.4235	1.0000
5	28	43	1496	2304	0.5408	1.0000
6	41	57	1483	2290	0.3419	1.0000
7	22	30	1502	2317	0.3812	1.0000
8	54	36	1470	2311	5.0281E-05	**2.4638E-03**
9	15	34	1509	2313	0.9226	1.0000
10	43	34	1481	2313	2.2864E-03	0.1120
11	39	69	1485	2278	0.7882	1.0000
12	29	31	1495	2316	0.0978	1.0000
13	36	44	1488	2303	0.1770	1.0000
14	34	39	1490	2308	0.1253	1.0000
15	37	76	1487	2271	0.9421	1.0000
16	34	54	1490	2293	0.5970	1.0000
17	25	45	1499	2302	0.7737	1.0000
18	33	62	1491	2285	0.8515	1.0000
19	33	38	1491	2309	0.1328	1.0000
20	28	29	1496	2318	0.0845	1.0000
21	35	51	1489	2296	0.4401	1.0000
22	27	73	1497	2274	0.9968	1.0000
23	33	27	1491	2320	9.6964E-03	0.4751
24	29	34	1495	2313	0.1680	1.0000
25	37	52	1487	2295	0.3718	1.0000
26	34	56	1490	2291	0.6611	1.0000
27	26	48	1498	2299	0.8081	1.0000
28	32	28	1492	2319	0.0189	0.9255
29	18	72	1506	2275	1.0000	1.0000
30	37	66	1487	2281	0.7955	1.0000
31	33	35	1491	2312	0.0767	1.0000
32	26	49	1498	2298	0.8316	1.0000
33	38	61	1486	2286	0.6183	1.0000
34	26	44	1498	2303	0.6920	1.0000
35	21	43	1503	2304	0.8882	1.0000
36	37	52	1487	2295	0.3718	1.0000
37	21	57	1503	2290	0.9927	1.0000
38	31	47	1493	2300	0.5162	1.0000
39	27	49	1497	2298	0.7905	1.0000
40	43	62	1481	2285	0.4046	1.0000
41	33	27	1491	2320	9.6964E-03	0.4751
42	29	44	1495	2303	0.5198	1.0000
43	21	49	1503	2298	0.9612	1.0000
44	31	41	1493	2306	0.2980	1.0000
45	35	47	1489	2300	0.3044	1.0000
46	31	38	1493	2309	0.2028	1.0000
47	24	46	1500	2301	0.8419	1.0000
48	25	51	1499	2296	0.9018	1.0000
49	27	74	1497	2273	0.9975	1.0000
50	26	45	1498	2302	0.7242	1.0000
